# Mitochondrial DNA Markers Reveal High Genetic Diversity but Low Genetic Differentiation in the Black Fly *Simulium tani* Takaoka & Davies along an Elevational Gradient in Malaysia

**DOI:** 10.1371/journal.pone.0100512

**Published:** 2014-06-18

**Authors:** Van Lun Low, Peter H. Adler, Hiroyuki Takaoka, Zubaidah Ya’cob, Phaik Eem Lim, Tiong Kai Tan, Yvonne A. L. Lim, Chee Dhang Chen, Yusoff Norma-Rashid, Mohd Sofian-Azirun

**Affiliations:** 1 Institute of Biological Sciences, Faculty of Science, University of Malaya, Kuala Lumpur, Malaysia; 2 School of Agricultural, Forest and Environmental Sciences, Clemson University, Clemson, South Carolina, United States of America; 3 Institute of Ocean and Earth Sciences, University of Malaya, Kuala Lumpur, Malaysia; 4 Department of Parasitology, Faculty of Medicine, University of Malaya, Kuala Lumpur, Malaysia; Auburn University, United States of America

## Abstract

The population genetic structure of *Simulium tani* was inferred from mitochondria-encoded sequences of cytochrome c oxidase subunits I (COI) and II (COII) along an elevational gradient in Cameron Highlands, Malaysia. A statistical parsimony network of 71 individuals revealed 71 haplotypes in the COI gene and 43 haplotypes in the COII gene; the concatenated sequences of the COI and COII genes revealed 71 haplotypes. High levels of genetic diversity but low levels of genetic differentiation were observed among populations of *S. tani* at five elevations. The degree of genetic diversity, however, was not in accordance with an altitudinal gradient, and a Mantel test indicated that elevation did not have a limiting effect on gene flow. No ancestral haplotype of *S. tani* was found among the populations. Pupae with unique structural characters at the highest elevation showed a tendency to form their own haplotype cluster, as revealed by the COII gene. Tajima’s D, Fu’s Fs, and mismatch distribution tests revealed population expansion of *S. tani* in Cameron Highlands. A strong correlation was found between nucleotide diversity and the levels of dissolved oxygen in the streams where *S. tani* was collected.

## Introduction

A wealth of biological diversity is associated with the broad range of abiotic and biotic factors typically inherent in altitudinal variation [1], [2]. Moreover, elevation can create barriers to gene flow in a wide variety of organisms, driving biological diversification [3]. Revealing patterns of variation from the level of the gene to the community is a first step in understanding the processes responsible for generating biodiversity [4], [5].

Black flies are ubiquitous inhabitants of streams and rivers over a wide range of elevations throughout the world. They are hypothesized to have originated in cool, mountainous areas [6]; consequently, they are an ideal group of insects for investigating the relation of genetic diversity to elevation. A total of 2151 species of black flies has been documented worldwide, with at least 75 species in Malaysia [7]. Female black flies are well known for their medical and veterinary importance [6], [8].

The most cytogenetically diverse black fly in the Oriental Region is *Simulium tani* Takaoka & Davies, with 11 known cytoforms [9], [10]. It was first described from Sungai Petani, a town in Kedah, a northern state of Peninsular Malaysia, bordering Thailand, and subsequently has been recorded from Thailand, Indonesia, and Vietnam [7], [11]. A molecular phylogeographical study using the mitochondrial cytochrome c oxidase subunit I (COI) gene revealed high genetic diversity and genetic differentiation in *S. tani* from different geographical regions of Thailand [12]. However, the genetic diversity and population structure of Malaysian isolates have not been characterized, although the type locality of *S. tani* is in Malaysia.

In Cameron Highlands, Malaysia, a monthly survey of black flies at five different elevations (0–1500 m above sea level) showed that *S. tani* was found at different frequencies at each elevation. *Simulium tani* was predominantly found at 0–300 m, 601–900 m and 1201–1500 m, but less prevalent at 301–600 m and 901–1200 m (personal observations). Pupae of *S. tani* at the highest elevation had unique structural characters (nearly smooth frons and slender gill filaments), whereas those at lower elevations had the typical condition (moderately tuberculate frons and thicker gill filaments). The pupal characters of the high-elevation population were associated with unique cytogenetic features, suggesting some genetic differentiation, although only a single cytoform (‘K’) is recognized in Cameron Highlands [10].

Given this morphological and chromosomal variation, the current study aimed to determine the intraspecific genetic diversity of *S. tani* and evaluate the degree of genetic differentiation along an elevational gradient, based on mitochondria-encoded COI and COII genes. Selected environmental factors associated with the population genetic structure of *S. tani* also were investigated. This fine-scale population genetic study complements the broad-scale study by Pramual et al. [12] to understand patterns of genetic variation in *S. tani*.

## Materials and Methods

### Ethics Statement

No national permissions were required for this study, which did not involve endangered or protected species. No specific permissions were required to access the study sites; the collections were made on public lands.

### Black Fly Specimens

**Figure 1:**
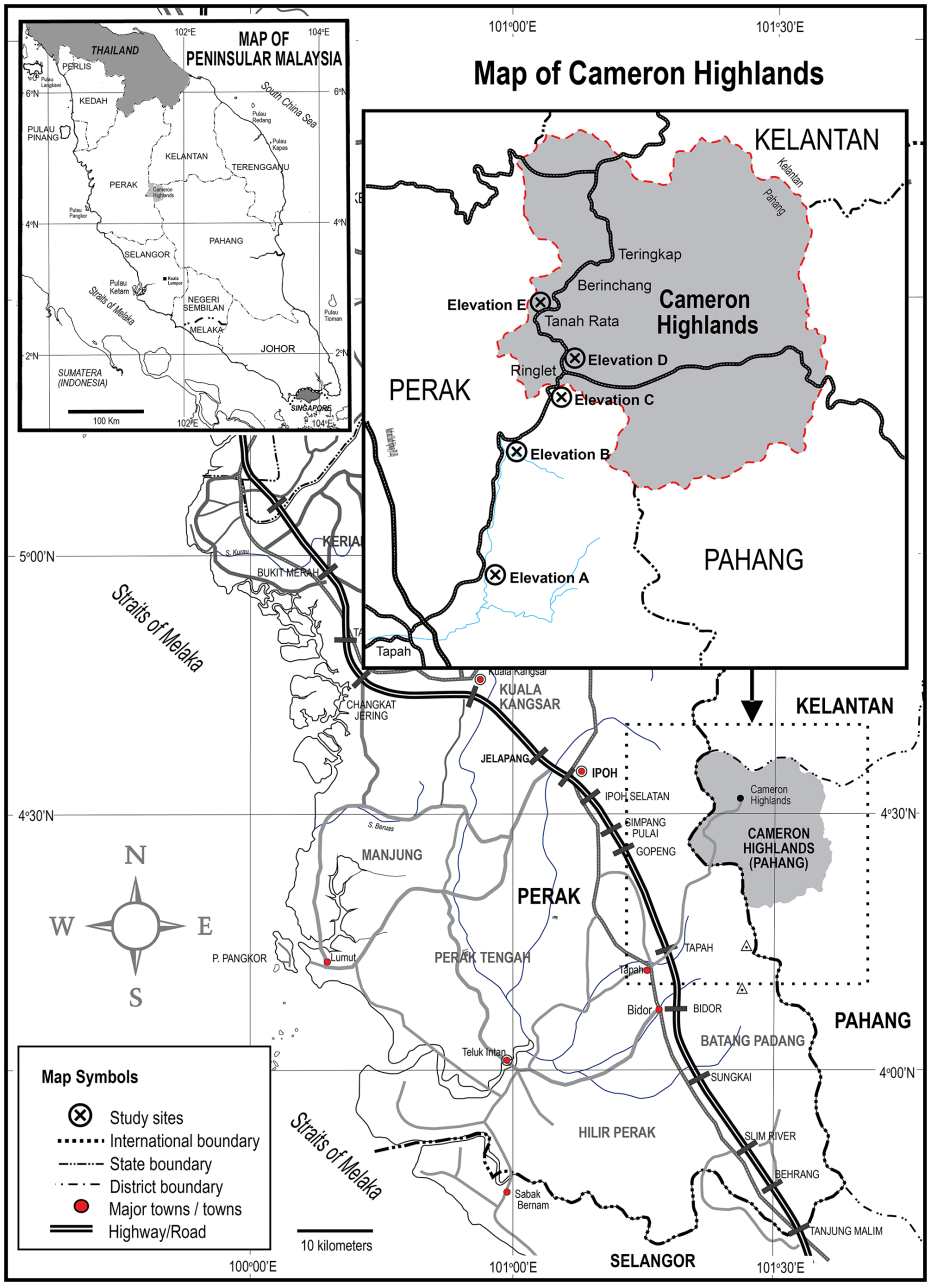
Sampling locations of *Simulium tani* at five elevations in Cameron Highlands, Malaysia.

Black fly pupae were sampled monthly in 2012 from five different elevations (A–E) in Cameron Highlands, Malaysia: A = 0–300 m, B = 301–600 m, C = 601–900 m, D = 901–1200 m, and E = 1201–1500 m (Figure 1). Pupae of *S. tani* were found every month at Elevations A, C and E; in February–April, June, July, October, and December at Elevation B; and only in February and December at Elevation D. Pupae were removed by hand from in-stream vegetation, placed in moistened 8-ml clear plastic tubes, and held in a cooler to allow emergence of adults, which then were identified using taxonomic keys [11]. Adults reared from all pupae discovered at Elevations B (n = 7) and D (n = 4) were used in analyses, whereas randomly selected individuals from larger samples collected at Elevations A (n = 21), C (n = 18), and E (n = 21) were analyzed.

The following stream characteristics were measured at each of the five collection sites during each of the 12 monthly visits: temperature, width, depth, current velocity, conductivity, dissolved oxygen, and pH (Table 1).

**Table 1:** Sampling locations and ecological parameters (mean ± standard error) for stream sites where *S. tani* was collected monthly in 2012 at five elevations (A–E) in Cameron Highlands, Malaysia.

Location	Altitude (m)	Latitude/Longitude	n^a^	Temperature (°C)	Width (m)	Depth (m)	Velocity (m/s)	Conductivity (m S)	DO^b^ (mg/L)	pH
**A**	235	N04°16′/E101°19′	21	24.48±0.29	4.31±1.70	0.18±0.02	0.02±0.00	0.17±0.01	6.68±0.28	6.99±0.11
**B**	337	N04°18′/E101°19′	7	22.99±0.24	1.70±0.85	0.15±0.02	0.02±0.00	0.36±0.11	7.20±0.04	7.10±0.10
**C**	711	N04°22′/E101°21′	18	20.73±0.46	2.32±0.94	0.11±0.06	0.02±0.00	0.44±0.09	6.05±0.06	7.18±0.01
**D**	1160	N04°26′/E101°22′	4	19.13±0.66	0.53±0.11	0.13±0.03	0.03±0.00	0.41±0.12	6.65±0.38	7.10±0.06
**E**	1405	N04°28′/E101°22′	21	18.00±0.24	2.57±0.92	0.11±0.04	0.03±0.01	0.31±0.12	7.17±0.20	6.85±0.07

a Number of specimens molecularly analyzed.

b DO = dissolved oxygen.

### DNA Extraction, Amplification, Purification, and Sequencing

DNA of *S. tani* was isolated successfully from all reared adult specimens subjected to analysis (n* = *71), using the i-genomic CTB DNA Extraction Mini Kit (iNtRON Biotechnology, Inc., Seongnam, South Korea). All extraction steps were performed according to the manufacturer’s instructions.

To resolve the phylogeographical and phylogenetic relationships of *S. tani* from different elevations in Cameron Highlands, 71 individuals were subjected to mitochondrial COI and COII amplifications. For comparison, amplifications of the nuclear encoded 18S ribosomal RNA (18S) and the 28S ribosomal RNA (28S) genes of *S. tani* were performed using a subset of 20 representative individuals. Preliminary data revealed no polymorphic sites in the 18S or 28S sequences of *S. tani* from different elevations, whereas COI and COII sequences were more valuable in resolving intraspecific relationships. Therefore, meaningful comparisons were made by using COI and COII sequences as the molecular markers.

Amplifications of the COI, COII, 18S, and 28S genes were performed in a final volume of 50 µL containing 0.5–1.0 µg genomic DNA, 25 µL of ExPrime Taq Master Mix (GENETBIO Inc., Daejeon, South Korea), and 10 pmol of each forward and reverse primer. Details of the polymerase chain reaction (PCR) primers are summarized in Table 2. PCR was carried out using Applied Biosystems Veriti 96-Well Thermal Cycler (Applied Biosystems, Inc., Foster City, CA, U.S.A.) The parameters of PCR amplifications of the COI, 18S, and 28S genes were 3 min at 94°C, followed by 35 cycles of denaturation at 94°C for 30 s; annealing at 55°C (COI), 59°C (18S), and 60°C (28S) for 30 s; extension at 72°C for 45 s, and a final extension at 72°C for 10 min. For COII, the cycling parameters were 2 min at 94°C, 45 s at 49°C, 45 s at 72°C, followed by 36 cycles of denaturation at 94°C for 30 s, annealing at 49°C for 45 s, extension at 72°C for 45 s, and a final extension at 72°C for 4 min [13].

**Table 2:** Primers used for amplification and sequencing of COI, COII, 18S, and 28S DNA sequences of *Simulium tani*.

Locus	Primer and sequence (5′-3′)	Amplicon size (bp)	Reference
COI	BCOI_F: GCA GGA GCT GGA ACA GGT TG	∼910	This study
	BCOI_R: TCC TAG GAA ATG TTG TGG GAA A		
COII	TL2-J-3034: ATT ATG GCA GAT TAG TGC A	∼810	[38]
	TK-N-3785: GTT TAA GAG ACC AGT ACT TG		
18S	B18S_F: TTT TAT GCA AGC CAA GCA CA	∼920	This study
	B18S_R: TGG GAA TTC CAG GTT CAT GT		
28S	B28S_F: GAA AAG GGA AAA GTC CAG CAC	∼890	This study
	B28S_R: CAC ATT TTA TGC GCT CAT GG		

The amplified fragments were electrophoresed on 2% agarose gels pre-stained with SYBR Safe (Invitrogen Corp., Carlsbad, CA, U.S.A.) The PCR products were purified with MEGAquick-spin PCR & Agarose Gel DNA Extraction System (iNtRON Biotechnology, Inc., Seongnam, South Korea). Purified PCR products were sent to a commercial company for DNA sequencing in both directions. Samples were sequenced using BigDyeH Terminator 3.1 Sequencing Kit and analyzed using an ABI PRISM 377 Genetic Analyzer (Applied Biosystems, Inc., Foster City, CA, U.S.A.).

### Data Analyses

Sequencing data were analyzed and edited using ChromasPro 1.7.6 (Technelysium Pty Ltd., Qld, Australia) and BioEdit 7.0.9.0 [14]. The sequences were preliminarily aligned using the CLUSTAL X program [15] and subsequently aligned manually. Representative sequences of COI (KJ636845–KJ636915), COII (KJ636916–KJ636958), 18S (KJ636959), and 28S (KJ636960) of *S. tani* were deposited in the NCBI GenBank. The COII and 18S sequences of *S. tani* were generated for the first time in this study, filling a void in the GenBank database.

The genetic diversity or haplotype networks of *S. tani* were analyzed using a median-joining algorithm [16] in the program Network 4.6. The aligned COI and COII sequences consisted of 831 bp and 684 bp, respectively. Multiple sequences of both COI and COII were concatenated to yield a total length of 1515 bp.

To access the genetic divergence of *S. tani* in both COI and COII genes, uncorrected (p) pairwise genetic distances were calculated using PAUP 4.0B10 [17].

To determine the level of genetic differentiation among populations, gene flow and genetic differentiation tests were performed with the program DnaSP 5.0 [18]. Haplotype diversity (Hd), nucleotide diversity (pi), genetic differentiation (F_ST_), and gene flow (Nm) values were obtained from these tests. The levels of genetic differentiation can be categorized as *F_ST_* >0.25 (great differentiation), 0.15 to 0.25 (moderate differentiation), and *F_ST_* <0.05 (negligible differentiation) [19]. The levels of gene flow can be categorized as Nm >1 (high gene flow), 0.25 to 0.99 (intermediate gene flow), and Nm <0.25 (low gene flow) [20]. Tajima’s D [21], Fu’s Fs [22], and mismatch distribution tests were performed with the program DnaSP 5.0 to test for changes in population size. Harpending’s raggedness index [23] and the R_2_ statistic of Ramos-Onsins and Rozas [24] also were determined. If expansion event is occurred, the time since expansion can be determined using mismatch calculator available at http://www.uni-graz.at/zoowww/mismatchcalc/. The expansion time was estimated based on the assumption of 12 generations a year for tropical black flies [12] and a divergence rate of 2.3% per million years for insect mitochondrial DNA [25].

The associations between genetic distance (*F_ST_*) and elevation difference (m) were examined using Mantel tests [26] implemented with the program IBD Web Service 3.23 [27]. Spearman rank-order correlations were performed with the statistical program SPSS 18 to determine associations between nucleotide diversity and stream characteristics at collection sites (i.e., temperature, width, depth, velocity, conductivity, dissolved oxygen and pH).

## Results

**Figure 2:**
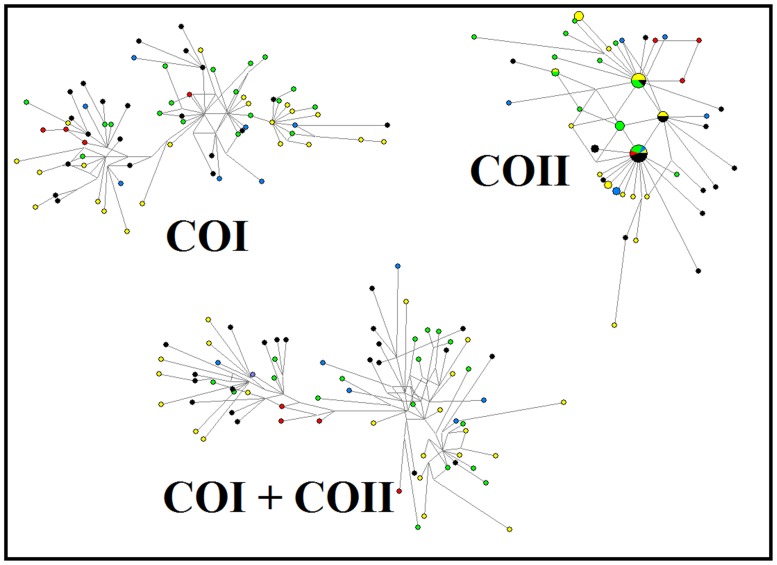
Median joining network of 71 taxa of the COI, COII, and COI+COII sequences from five different populations of *Simulium tani* in Cameron Highlands. Each haplotype is represented by a circle. Relative sizes of the circles indicate haplotype frequency. Circles of the same colour represent haplotypes from the same population (yellow = Elevation A, blue = Elevation B, green = Elevation C, red = Elevation D and black = Elevation E).

A statistical parsimony network of 71 individuals aligned as 831 characters of the COI gene and 684 characters of the COII gene revealed high levels of genetic diversity among populations of *S. tani* in Cameron Highlands. A total of 71 and 43 haplotypes were inferred from the COI and COII genes, respectively. For concatenated sequences, a total of 1515 characters of both COI and COII genes revealed 71 haplotypes. No ancestral haplotype of *S. tani* was found among the populations. The median-joining network of the COI and COI+COII genes demonstrated a lack of clear separation among populations, and the haplotypes were well dispersed across all study sites. However, based on the COII gene, the population at Elevation C (green) and the population with structurally unique pupae at Elevation E (black) showed a slight tendency to form different clusters (Figure 2).

COI demonstrated higher resolving power (0.12–1.81%) than did COII (0.00–1.17%) or COI+COII (0.13–1.32%) for genetic distance (Tables 3, 4).

**Table 3:** Percentage of uncorrected “p” distance matrix between elevations (A-E) based on COI (lower left matrix) and COII (upper right matrix) DNA sequences of *Simulium tani* from Cameron Highlands, Malaysia.

	A	B	C	D	E
**A**	–	0.00–0.88	0.00–1.17	0.00–0.88	0.00–1.02
**B**	0.48–1.44	–	0.00–1.02	0.15–0.73	0.00–1.17
**C**	0.12–1.56	0.24–1.56	–	0.00–0.88	0.00–1.17
**D**	0.24–1.44	0.36–1.32	0.24–1.32	–	0.15–0.58
**E**	0.12–1.68	0.24–1.81	0.12–1.81	0.12–1.56	–

**Table 4:** Percentage of uncorrected “p” distance matrix between elevations (A–E) based on COI+COII DNA sequences of *Simulium tani* from Cameron Highlands, Malaysia.

	A	B	C	D	E
**A**	–				
**B**	0.20–1.12	–			
**C**	0.13–1.25	0.20–1.12	–		
**D**	0.13–1.12	0.26–0.99	0.20–0.99	–	
**E**	0.13–1.32	0.33–1.19	0.13–1.32	0.12–0.20	–

In total data estimates, the COI gene revealed higher haplotype diversity (1.000) and nucleotide diversity (0.0089) than did the COII gene, which had 0.9602 for haplotype diversity and 0.0045 for nucleotide diversity (Table 5). The COI and COI+COII data showed that all sequences from the five populations were unique in having 1.000 for haplotype diversity. Given that all populations showed maximum haplotype diversity, the assessment of genetic diversity of *S. tani* was based on nucleotide diversity. The COI, COII, and COI+COII sequences produced consistent results, with the highest level of nucleotide diversity at Elevation B and the least diversity at Elevation D. Generally, the levels of nucleotide diversity in descending order were as follows: Elevation B (301–600 m)>Elevation E (1201–1500 m)>Elevation A (0–300 m)>Elevation C (601–900 m)>Elevation D (901–1200 m).

**Table 5:** Haplotype diversity (Hd), nucleotide diversity (pi), Tajima’s D (D) and Fu’s Fs (Fs) tests based on COI, COII, and COI+COII DNA sequences of *Simulium tani* from Cameron Highlands, Malaysia.

	COI				COII				COI+COII			
	Hd	pi	D	Fs	Hd	Pi	D	Fs	Hd	pi	D	Fs
**A**	1.000	0.0090	−1.3545	−16.3920	0.9476	0.0043	−1.0809	−8.1830	1.000	0.0069	−1.3229	−13.1220
**B**	1.000	0.0099	−0.9275	−1.9350	0.9524	0.0050	−0.3543	−1.9290	1.000	0.0077	−0.7861	−1.3000
**C**	1.000	0.0081	−1.3870	−13.5740	0.9412	0.0043	−1.2459	−6.1040	1.000	0.0064	−1.3928	−10.5970
**D**	1.000	0.0058	−0.1536	−0.5690	1.000	0.0034	0.6501	−1.6220	1.000	0.0047	0.1091	−0.0650
**E**	1.000	0.0091	−1.1081	−16.2520	0.9429	0.0044	−1.8339	−9.8890	1.000	0.0070	−1.4211	−12.9780
**Total**	1.000	0.0089	−1.8282	−34.0390	0.9602	0.0045	−2.1908	−51.6800	1.000	0.0069	−2.0617	−94.9890

The study revealed a relatively low level of genetic differentiation among the five populations. The majority of the population pairs showed *F_ST_* <0.05, indicating low differentiation. Moderate differentiation (*F_ST_* >0.15), evidenced by the COI+COII genes, was found between Elevations A and D. Overall, however, no significant differentiation was observed among populations (*P*>0.05). High levels of gene flow occurred among populations, as evidenced by the high value of Nm 8.38 in the COI+COII genes (Table 6).

**Table 6:** Genetic differentiation (F_ST_) and gene flow (Nm) based on COI+COII DNA sequences of *Simulium tani* from Cameron Highlands, Malaysia.

Population 1	Population 2	COI+COII	
		F_ST_	Nm
A	B	−0.0024	−213.13
A	C	0.0075	66.57
A	D	0.1510	2.81
A	E	0.0307	15.81
B	C	−0.0215	−23.79
B	D	0.1054	4.24
B	E	0.0067	74.52
C	D	0.1292	3.37
C	E	0.0589	7.99
D	E	0.1113	3.99
		**Total Nm**	8.38

**Figure 3:**
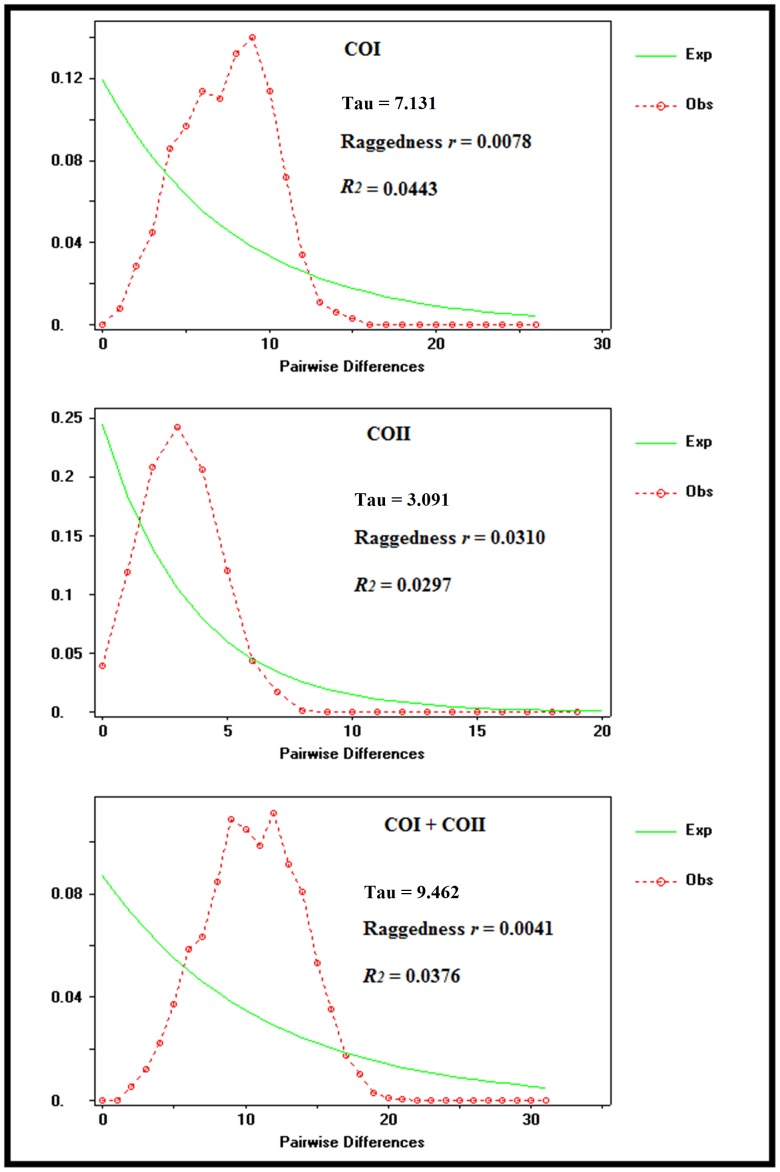
Observed and expected mismatch distributions for *Simulium tani* in Cameron Highlands, Malaysia, based on COI, COII, and COI+COII sequences.

The median-joining network of COI, COII, and COI+COII revealed “star-like” networks, suggesting population expansion. The unimodal mistmatch distributions, low values of the Raggedness index (*P*>0.05) and R_2_ statistic (*P*<0.05) from mismatch distribution tests (Figure 3), along with the negative values of Tajima’s D and Fu’s Fs (*P*<0.05) (Table 5), further supported the hypothesis of population expansion of *S. tani* in Cameron Highlands. The expansion times are estimated to be 200, 000 (COI), 370,000 (COII) and 270,000 (COI+COII) years ago.

**Figure 4:**
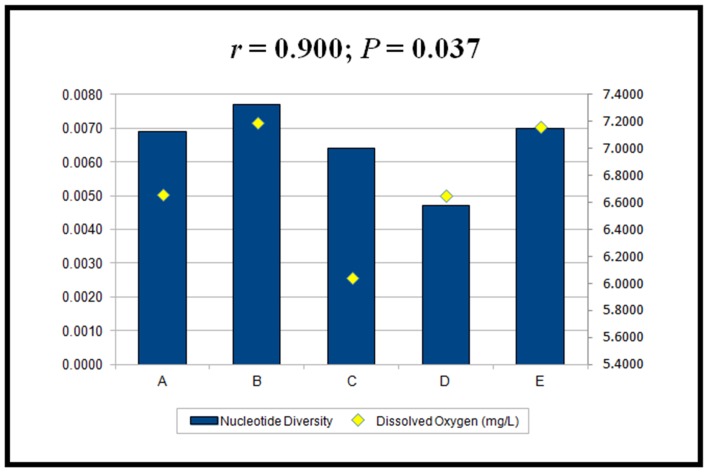
Spearman rank-order correlation between the nucleotide diversity of *Simulium tani* and the levels of dissolved oxygen in Cameron Highlands, Malaysia.

A Mantel test revealed no significant association between genetic distance (*F_ST_*) and elevation (m), suggesting that isolation by distance did not have a limiting effect on gene flow. Spearman rank-order correlation revealed that the nucleotide diversity (COI and COI+COII genes) of *S. tani* was positively correlated with the levels of dissolved oxygen in the stream water (*r = *0.900; *P* = 0.037) (Figure 4). No significant association (*P*>0.05) was found for the other six stream variables (i.e., temperature, width, depth, velocity, conductivity, and pH).

## Discussion

The median-joining network demonstrated a high level of genetic diversity for populations of *S. tani* in Cameron Highlands, with 71 unique haplotypes among 71 examined individuals that did not share the same ancestral haplotype. The high level of genetic variation in *S. tani* was corroborated by the high degree of haplotype diversity (1.0000) and nucleotide diversity (0.0058–0.0099) and by the uncorrected “p” distance matrix (0.12–1.81%), based on the COI gene. The wide-scale study by Pramual et al. [12] also revealed high genetic diversity of *S. tani* in Thailand, with 121 unique haplotypes from 147 individuals and haplotype diversity and nucleotide diversity of 0.8570–1.0000 and 0.0030–0.0155, respectively. Based on haplotype frequencies, the genetic diversity of *S. tani* in the present study was greater than that previously described in Thailand for *S. tani* (by 1.21 fold), *S. feuerborni* Edwards (2.03 fold), and *S. siamense* Takaoka & Suzuki (3.54 fold) [12], [28], [29]. The population expansion of *S. tani* occurring in the mid Pleistocene agreed with the previous findings in Thailand [12]. However, the Thai *S. tani* has older expansion (500, 000 years) than Malaysian *S. tani* (200, 000–370,000 years).

A fine-scale study of *Simulium gravelyi* Puri in India revealed greater genetic diversity at high elevations (>1500 m) than at low elevations (<300 m) [30]. Our study, however, demonstrated that the degree of genetic diversity was not in accordance with an altitudinal gradient. The greatest nucleotide diversity was observed at 301–600 m and the least at 901–1200 m. These differences might reflect differences in characteristics of the streams where the black flies develop. A strong linear relation was found between nucleotide diversity and dissolved oxygen. The significance of this relationship is not known. However, the distributions of black flies, from molecular forms and cytotypes to full species, typically are associated with environmental variables, such as pH, stream size, and water temperature [31], [32].

Moreover, the role of anthropogenic disturbances in structuring the current genetic variability in *S. tani* in Cameron Highlands cannot be excluded. Over the years, pristine montane forests in Cameron Highlands have been cleared for intensive agricultural activities [33]. Destruction of riparian vegetation, siltation, and organic pollution associated with agriculture affect in-stream oxygen levels and the resident black flies [6], [31]. Pesticide contamination also has occurred in the watercourses of Cameron Highlands as a consequence of farming practices [34]. Hence, the black flies in Cameron Highlands might have experienced selection pressure from factors associated with agriculture, such as pesticides, leading to the observed high genetic variability.

A population model for mosquitoes [35] showed that the populations become smaller with increasing elevation, thereby reducing the degree of genetic diversity. This model, however, does not fit the data on the abundance and genetic variability of *S. tani* in Cameron Highlands. The monthly sampling data indicated low numbers of *S. tani* at Elevations B and D but high numbers at Elevations A (low elevation), C (mid-elevation) and E (high elevation). Additional sampling at higher elevations (>1600 m) throughout the year did not reveal *S. tani* (unpublished data). The relatively small samples from Elevations B and D in 2012 and 2013 suggested that these two sites might support temporary, rather than self-sustaining, populations. Although the sample sizes for Elevations B and D were not ideal for measuring the haplotype frequencies in total data estimates, the haplotype diversity and nucleotide diversity revealed by the COI and COII data were not influenced by the total number of individuals along the elevational gradient. In addition, a data set excluding the specimens from Elevations B and D was evaluated; the results were in concordance with the five-elevation data set, indicating low genetic differentiation and high gene flow in *S. tani* (results not shown).

Previous studies have revealed the importance of elevation as a barrier to gene flow for black flies [28], [36], [37]. In the present study, however, low levels of genetic differentiation and high levels of gene flow were detected among populations of *S. tani* in Cameron Highlands. Isolation by distance did not have a limiting effect on gene flow; no significant correlation was found between genetic distance and elevation. Hence, elevation is unlikely to be a physical barrier to gene flow for *S. tani*. The weakly formed haplotype cluster (COII gene) for the population of *S. tani* with distinct pupal features reflects the similarly weak, but distinct, cytogenetic differentiation previously reported [10] for *S. tani* in Cameron Highlands. The molecular results, thus, support the conclusion from cytogenetic data that a single taxonomic entity (Cytoform ‘K’) exists in Cameron Highlands [10]. The lack of elevational barriers to gene flow might be characteristic of *S. tani* across its range; previous studies [9], [12] have found *S. tani* only within the elevational range (0–1500 m) examined in Cameron Highlands. In Thailand, where high levels of genetic differentiation at both chromosomal and molecular scales have been found across regions, the lowlands and Gulf of Thailand have been suggested as physical barriers to gene flow [9], [12].

In conclusion, the present study–the first molecular phylogeographical study of Malaysian black flies–revealed high levels of genetic diversity but low levels of genetic differentiation in *S. tani* across an elevational gradient. Congruent results from cytogenetic, morphological, and molecular analyses indicating slight differences in the high-elevation population of *S. tani* suggest that applying all three analytical approaches provide a powerful means of unfolding the biodiversity hidden at multiple scales in black flies.
